# Biometric technology in “no-gate border crossing solutions” under consideration of privacy, ethical, regulatory and social acceptance

**DOI:** 10.1007/s11042-020-10266-0

**Published:** 2020-12-29

**Authors:** Susanne Binder, Andrea Iannone, Chad Leibner

**Affiliations:** 1 MultiMedia and Visions Lab, London, E1 4NS UK; 2 Queen Mary University of London, London, UK; 3 CyberEthicsLab, Via Antonio Salandra, 18, 00187 Rome, Italy; 4 Ministry of Public Security, Ba’alei Hamelacha 41, 72558 Ramle, Israel

**Keywords:** Biometrics, Border security, Impact assessment, Ethics, Privacy

## Abstract

Biometric technologies have become the main focus in the design of state-of-the-art border security solutions. While respective research in the field of multimedia vision has been centred around improving quality and accuracy of identity recognition, the impact of such technologies upon society and legal regulations still remains a topic unaddressed, specifically within the engineering community. Research in technology can and in some respect must include collaboration with social sciences and social practice. Building on participation in the EU funded research project PERSONA [18] (Privacy, Ethical, Regulatory and SOcial No-gate crossing point solutions Acceptance), authors of this paper look at the challenges associated with biometrics-based solutions in no-gate border crossing point scenarios. This included the procedures needed for the assessment of their social, ethical, privacy and regulatory acceptance, particularly in view of the impact on both, the passengers and border control authorities as well as the potential pitfalls of biometric technology due to fraudulent activities. In consultation with the collaborating border control authorities, the paper reports on the formal assessment of biometric technologies for real-world acceptance to cope with the increasing demand of global travellers crossing state borders.

## Introduction

**Fig. 1:**
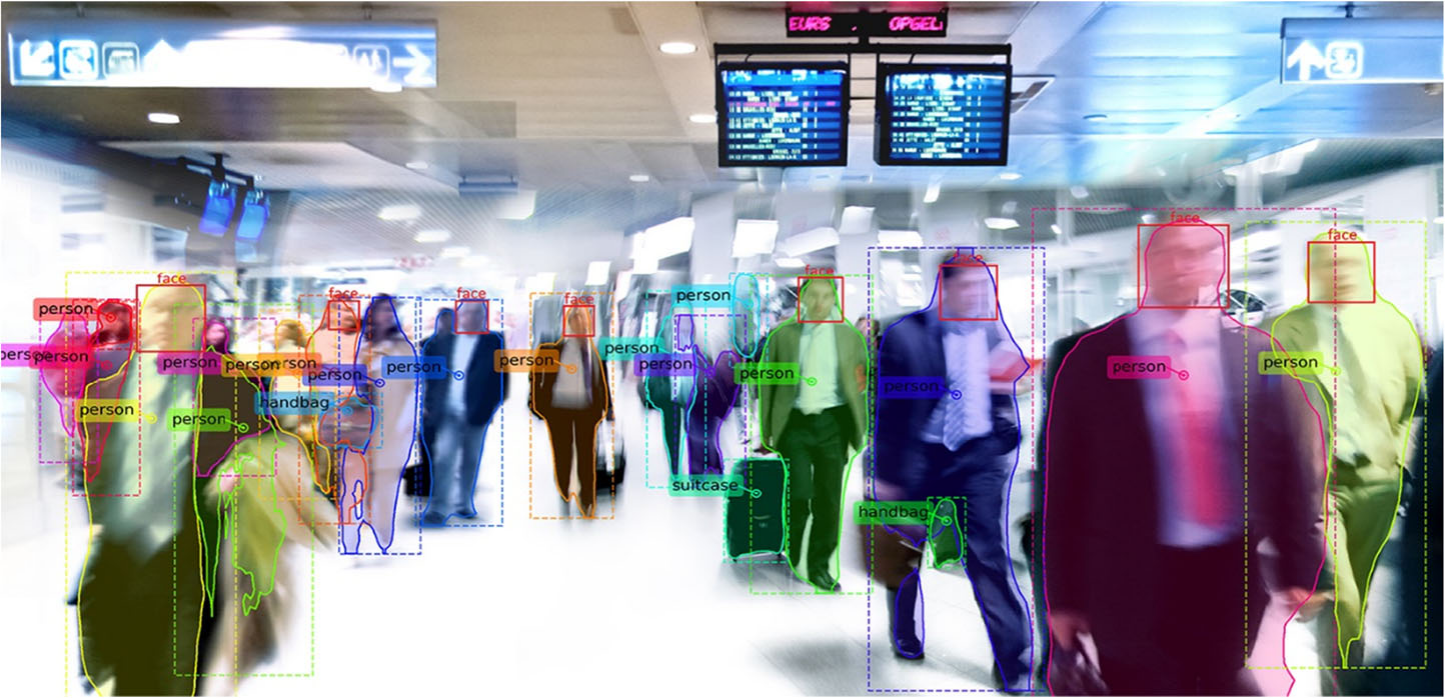
Use of computer vision technologies to capture individuals on their way to the departure gate by localising their facial features and comparing them to existing data base entries

The arising need at EU border crossings for efficient processing of an ever-increasing number of travellers calls for more flexible, automated and scalable “no-gate” border security solutions. A “No-Gate Border Crossing Point Solution” is understood to be a method which enables travellers to cross borders without being slowed down by physical checks, based on automated risk-assessment and/or biometric verification processes. While the use of biometric technology offers a promising pathway towards achieving seamless border crossing experience for the majority of travellers, the often-overlooked potential impact of its deployment has yet to be further investigated in order to be fully understood. The challenges of biometric technology development are thus reaching beyond improved accuracy in recognition. This method will likely comprise state-of-the-art technology in the shape of an information system and a network of sensors capable of detecting, recording, processing, and storing data which would otherwise have to be collected by traditional, more time-consuming means. This type of technology might be deployed in the vicinity of border crossing points, e.g., on the traveller’s way to the departure gate as shown in Fig. 1 [18], and/or it could be mobile/remote and perform checks on travellers along their journey, e.g., as early as “on the plane/train” before arrival or at the traveller’s home before journey commence, via remote online data-collection. It is foreseeable that such new technologies will stand ready to be implemented in the very near future to allow for seamless crossing of borders and security checks for the vast majority of travellers who meet the conditions of entry while ensuring that those who do not fulfil such conditions are refused entry.

**Fig. 2:**
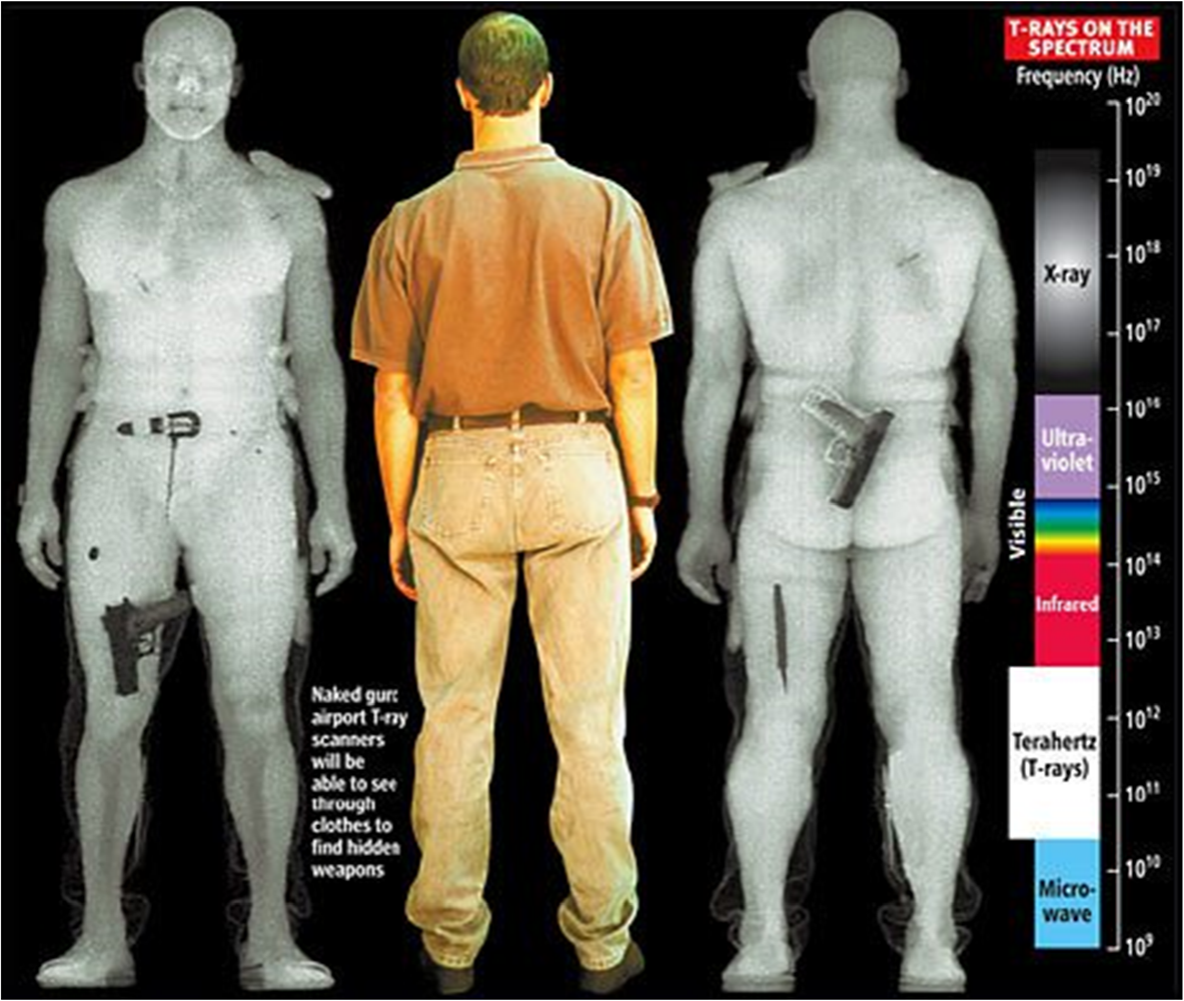
Images generated by a full-body scanner which displays a traveler’s body in a manner that may compromise privacy

Familiar, already existing technologies that could act as “components” in a No-Gate Border Crossing Point Solution are, e.g., cameras, body scanners, facial recognition methods, material detection methods, etc. Some of these existing technologies have been considered controversial due to their perceived intrusive nature as presented in Fig. 2 [5] which illustrates how images generated by a full-body scanner can potentially display the scanned traveller’s anatomy in unnecessarily intrusive detail.

Even though useful from a technical point of view, privacy should not be compromised, and ethical aspects should be taken into account. The H2020 project PERSONA (Privacy, Ethical, Regulatory and SOcial No-gate crossing point solutions Acceptance) aims at providing for respective authorities an assessment tool capable of appropriately evaluating such technology under aspects of data protection, privacy, and social acceptance. Inspired by the participation in PERSONA, authors look at ways to incorporate societal concerns into the very design and technical research process. This paper demonstrates on the example of relevant border security technology how metrics considered in the PERSONA impact assessment method can be adopted by or inform investigative technological thinking, where a positive evaluation result under the impact assessment method proposed by PERSONA forms one of the goals, and where interdisciplinary collaboration is being fostered.

The remainder of this paper is structured as follows: in Section 2, an overview of the applied biometrics in state border control is presented along with examples of test pilots concerning the potential deployment of no-gate border control through-tunnels. In addition, an outline of the challenges faced by the border control in evaluating various biometric features is also discussed. The PERSONA impact assessment study is presented in Section 3 followed by a review of ethical impact assessment in Section 4. Conclusions and future objectives are contained in Section 5.

## Biometrics technology in “no-gate” border crossing solutions

In Computer Science, the term “biometric technology” refers to the automated recognition of an individual by their biometrics, i.e. their associated unique anatomical features (e.g., iris, retinal, face, fingertip) or behavioural traits (e.g., gait, voice, keystroke, and signature), or more typically by the combination of them [17]. Biometrics are extracted using computer vision and digital analysis of biological characteristics captured via camera/sensor, increasingly providing a more secure and convenient way for personal authentication than alternative methods that rely on memorised data (e.g., passwords) or objects (e.g., ID cards).

**Fig. 3:**
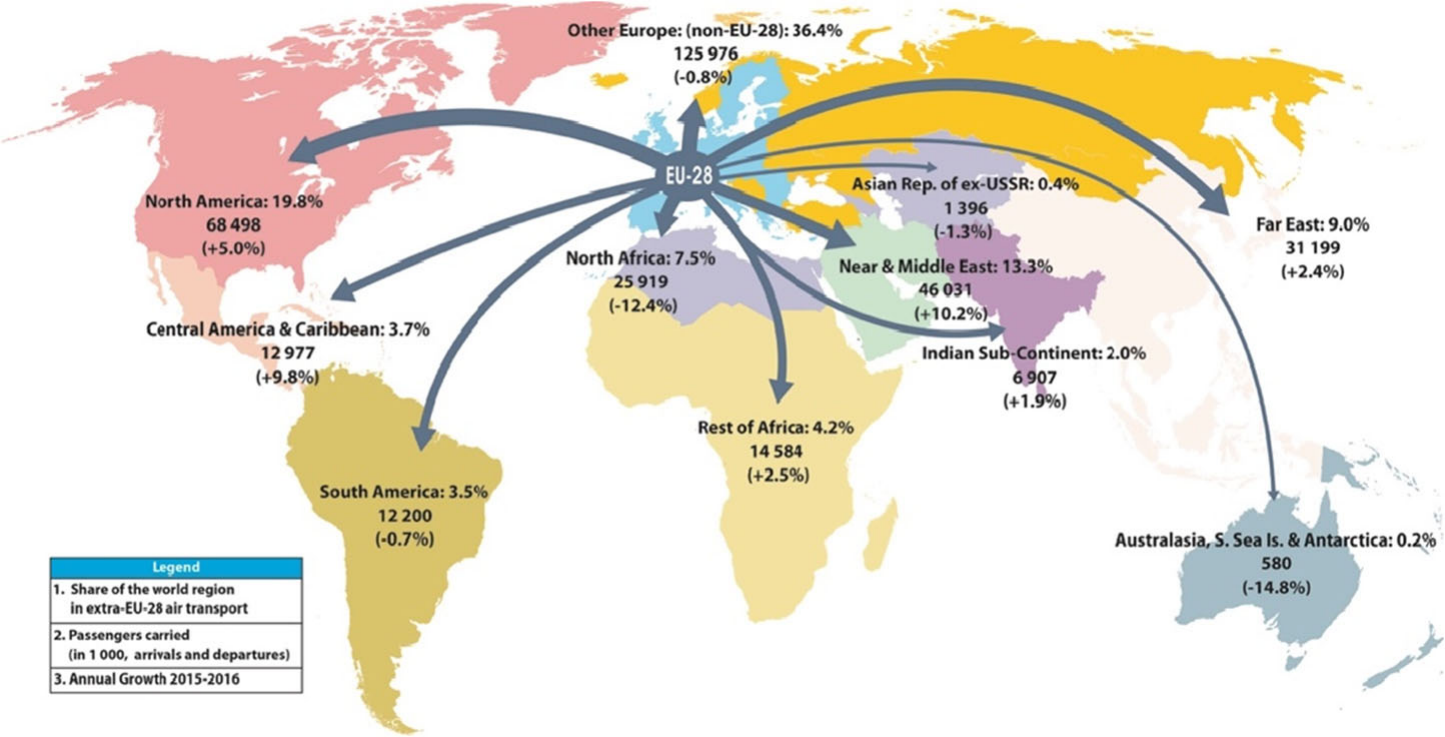
Global travel map of EU residents

Globalisation combined with an increasing level of average living standards have, over the last few decades, led to a continuous rise in international travel for both, business and leisure purposes. The European Agency for the Management of Operational Cooperation at the External Borders of the Member States of the European Union issued statistics stating that there were 672.3 million international tourist arrivals in 2018 alone [4]. Equally, the number of international itineraries undertaken by EU citizens is rapidly growing with every year (see Fig. 3 [4]), placing mounting pressure on the everyday handling of border checks.

**Fig. 4:**
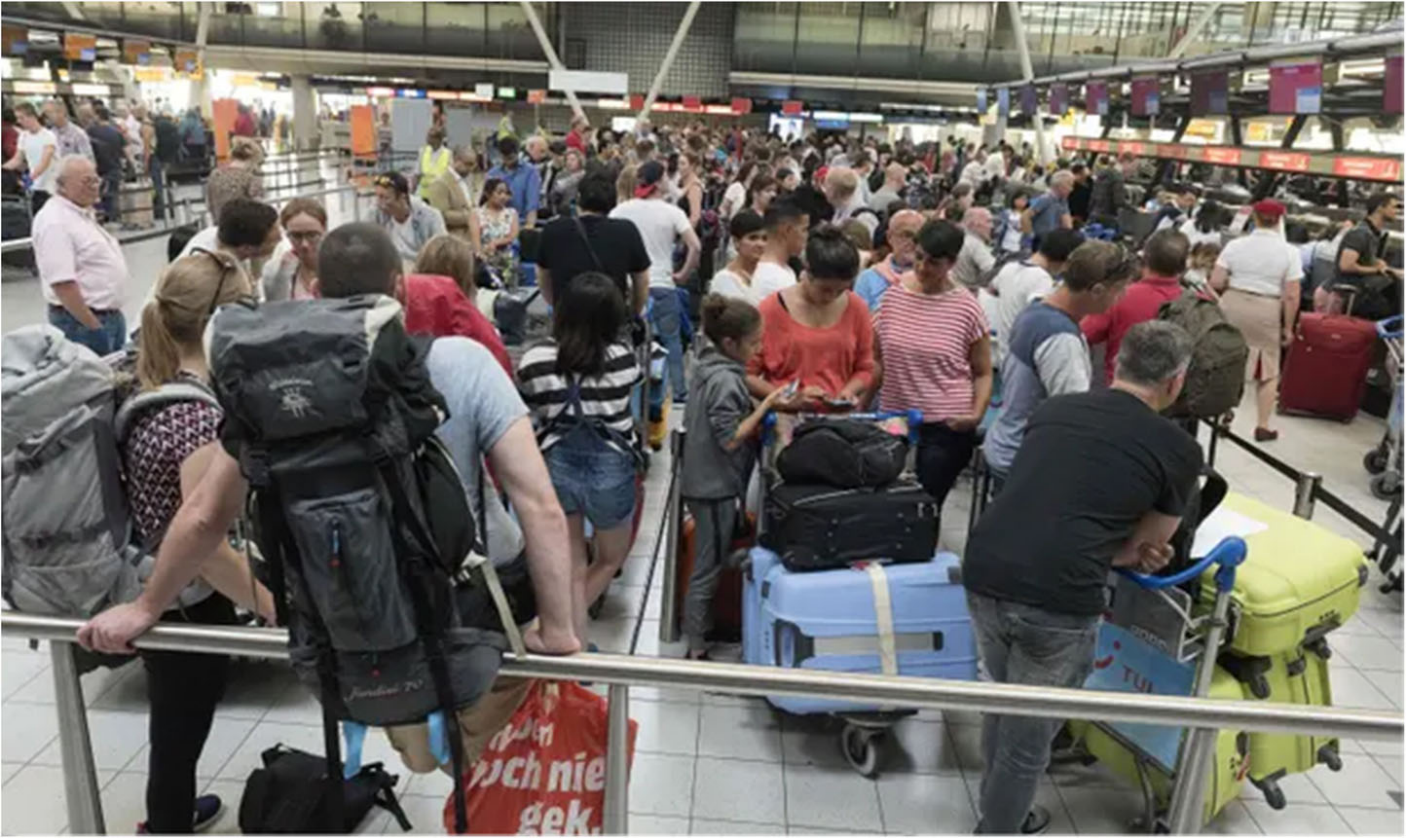
Passengers queuing for over 1 h due to border check delays at a Spanish airport, highlighting why speedier ways of processing at border crossing points are sought

In parallel, acute security/safety risks such as terrorism, people trafficking, and as now demonstrated through the covid-19 pandemic, spread of disease call for tighter and more thorough control measures for enhanced protection of citizens. As a result, border control authorities have to process a higher amount of more elaborate checks within an ever shorter time frame under dwindling resources, despite all efforts often leading to problematic congestion as shown in Fig. 4 [22].

In response, border authorities seek the deployment of flexible, automated and scalable “no-gate” border security solutions which (i) facilitate management of continuous high-volume or erratically changing quantities of passenger traffic; (ii) allow to spontaneously implement temporary extra security components at short notice if so required; (iii) minimise disruption for the traveller. However, the intensive use of qualifying technologies bears the risk of disproportionally invading people’s privacy; societal and political acceptance of technologies for contactless border security solutions should be precondition to their application.

**Fig. 5:**
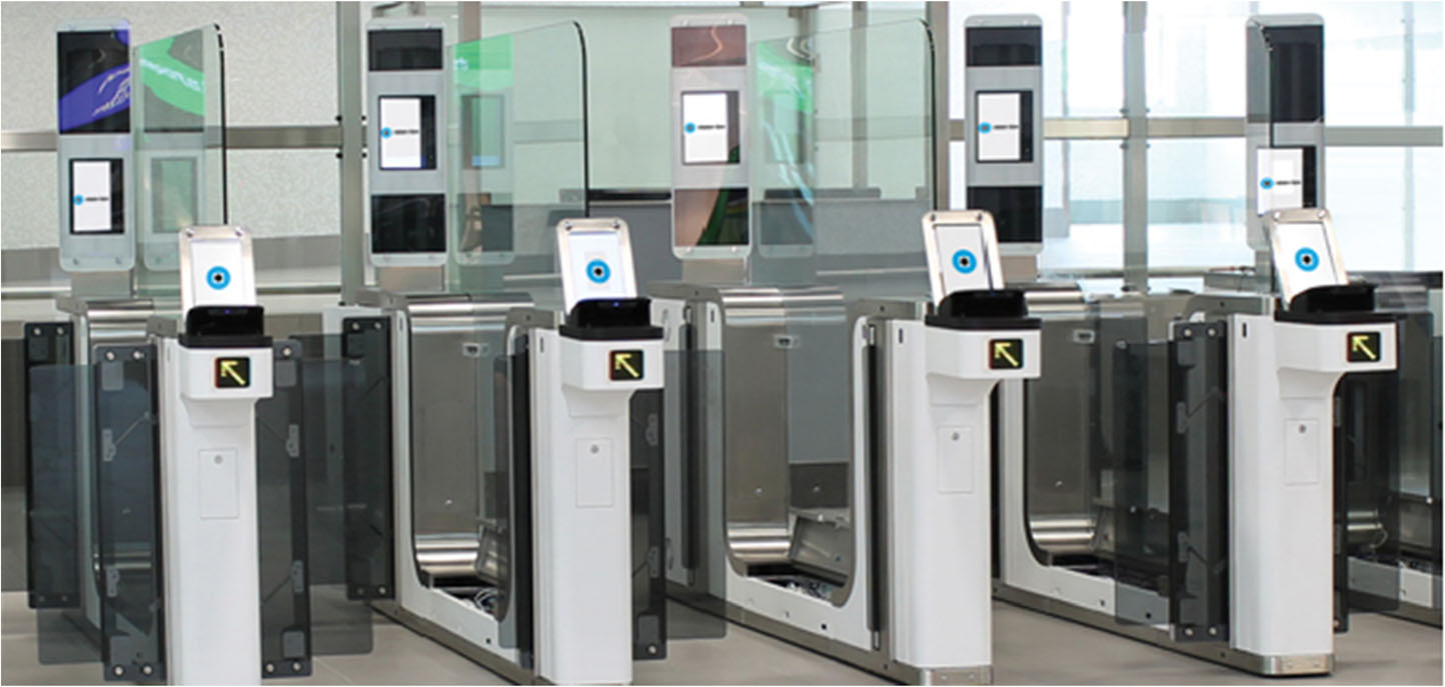
E-Gates for automated border control

As a means for reliable and highly accurate personal identification, biometric recognition technology is already being used in a wide range of government and commercial applications, including in international border crossing solutions, e.g., at international airports, effectively cutting passenger processing time and speeding up passenger flow. Examples for such common applications are e-passport checking with live photo and fingerprint reading as shown in Fig. 5 [3].

New generation border security technology goes further in combining the detection of different anatomical features in order to increase accuracy in identification while the steps for data gathering (e.g. scanning) are executed “on the go” to create a more seamless border crossing experience for the individual [19].

**Fig. 6:**
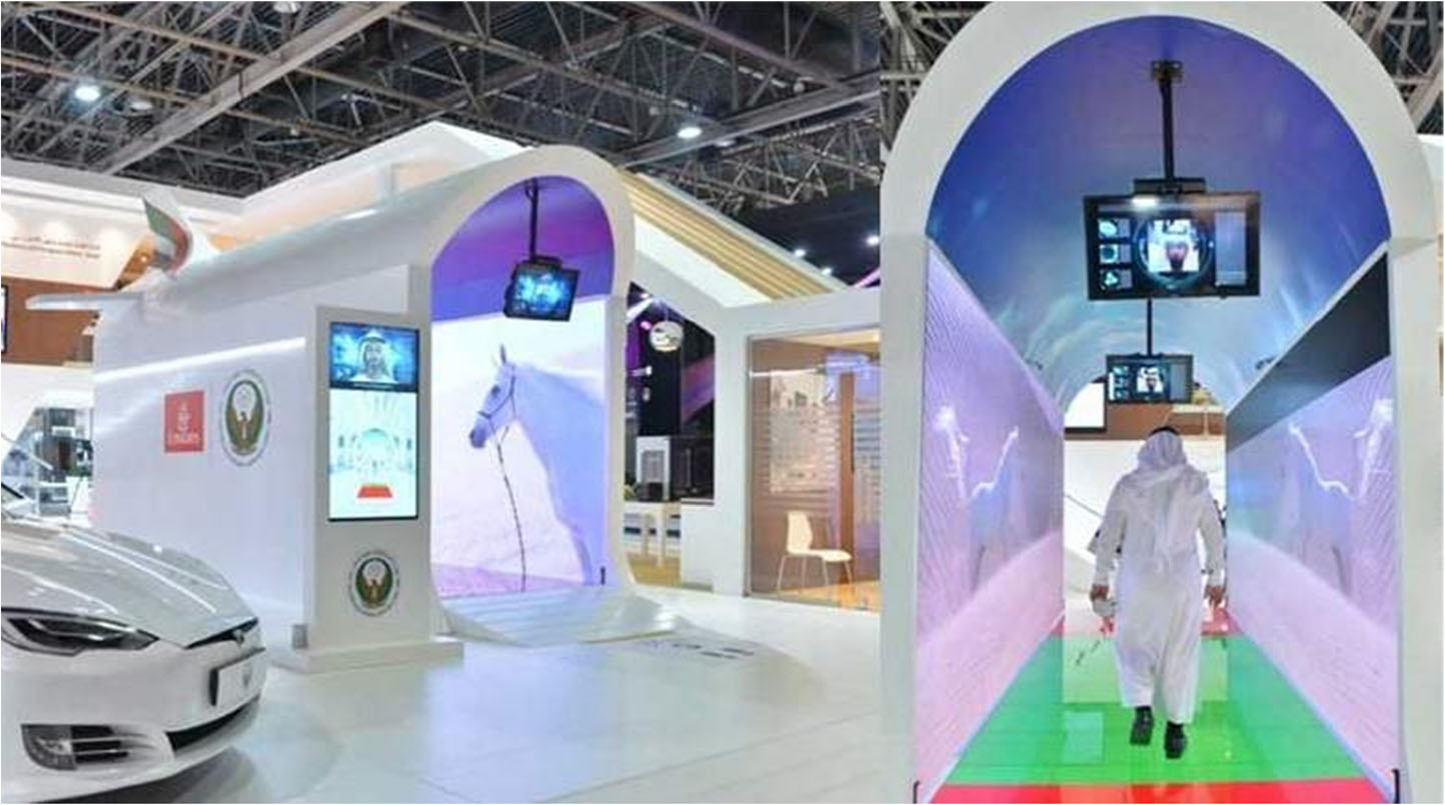
Smart tunnel presentation at gitex technology week

In October 2017, a Smart Tunnel system was presented at the Gitex Technology Week at Dubai World Trade Centre: a tunnel construction equipped with over 80 hi-tech cameras producing high-quality images funnels passengers to their destinations (e.g., a departure gate). Identification takes place during walk-through via a multi-scanning process of the individual’s biometric features (see Fig. 6) [3]. As passengers walk through, they can be identified through biometric technology which is scanning their face or iris using cameras while they are in motion. Apart from biometric recognition, the tunnel features a Full Body Scanner (FBS) which is conventionally not used in Dubai airports as this type of scanning violates Dubai ethics code [10]. For passengers who provide their details remotely via mobile app prior to the arrival, no human intervention or passport checking is needed during border crossing.

**Fig. 7:**
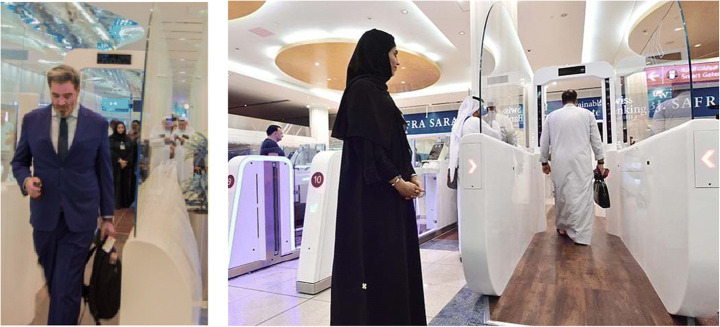
Smart tunnel at the DXB emirates terminal 3

One year on, from October 2018, a similar system has been trialled at Dubai International Airport (DXB) Emirates Terminal 3 (see Fig. 7). The process was dubbed “biometrics path” and was eventually linked with the General Directorate of Residence and Foreigners in Dubai’s (GDRFA’s) Smart Tunnel project.

The Smart Tunnel is not enclosed, resembling a longer version of the Smart Gates. The system uses facial and iris recognition technology, AI and machine learning. The objective is to allow passengers to smoothly pass through the tunnel in just a few seconds without the need for passport stamping or any other human intervention.

### Traditional challenges of biometrics technology

**Fig. 8:**
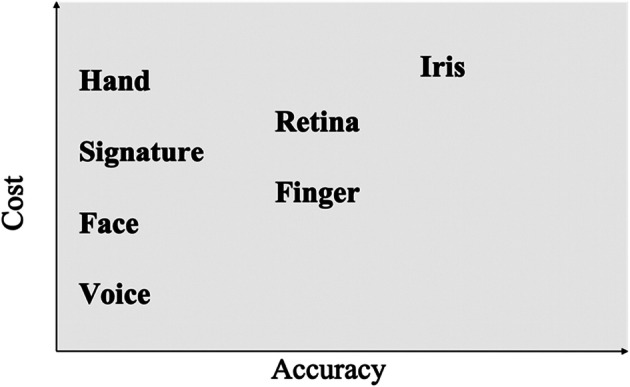
Biometric measures - relative cost and accuracy

Compared to passwords and tokens, biometric authentication methods can enhance user convenience but are also both, technically more complex and costly. Figure 8 compares the relative cost and accuracy of the different biometric measures [16].

**Fig. 9:**
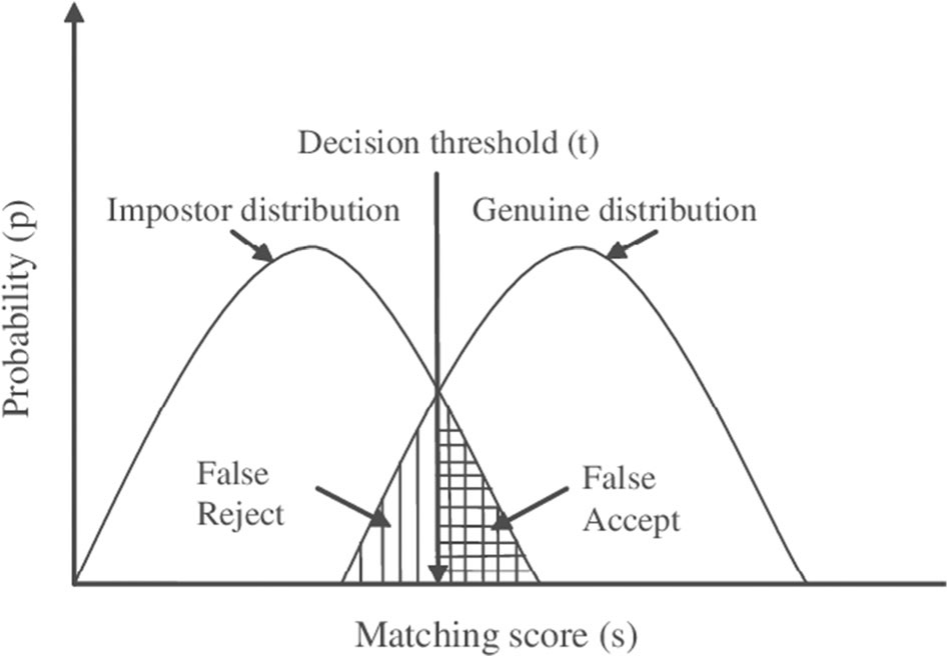
Genuine and impostor score distributions for a typical biometric matcher

Biometric pattern systems must determine how closely a presented biometric characteristic matches a stored characteristic. A biometric system typically has to navigate problems related to non-universality of biometric (failure to enrol rate), limited degrees of freedom (finite error rate), large intra-class variability, and spoof attacks (system security). In view of the error types, these can be either false accepts or false rejects, whereby the default target decision threshold is located at the point of equal error rate, as illustrated in Fig. 9 [2] but would, where tight security is required, be placed in favour of false rejects.

**Fig. 10:**
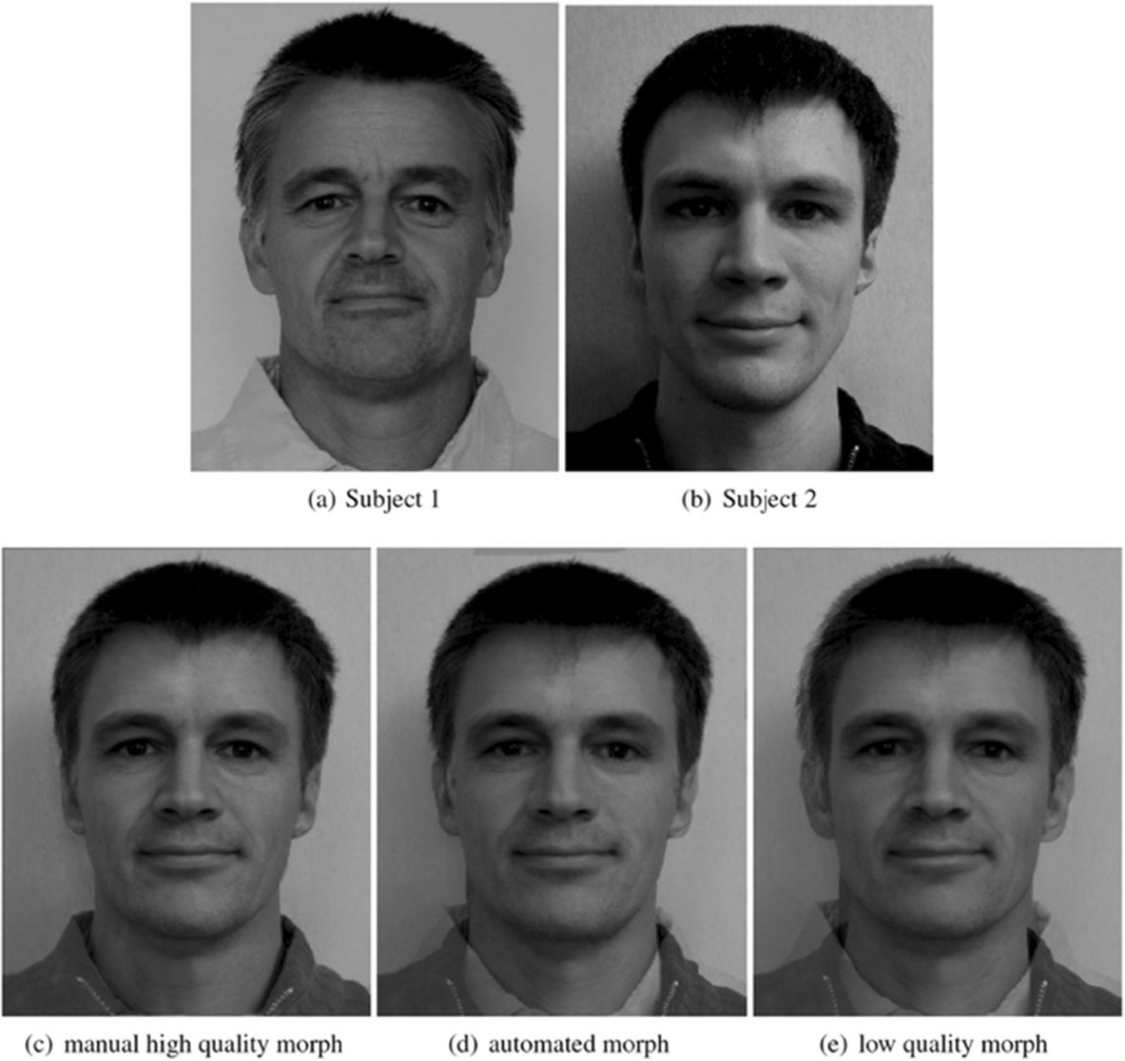
Three morphed facial image versions (row below) of two different persons (row above)

For its reputation of being relatively reliable and accurate, the detection of some biometric features such as “face” [20] possesses, often unexpected to the layman, a surprisingly high scope for error; while allowances have to be made for common fluctuations in appearance (e.g., more “puffy” in the morning, body weight related changes, ageing, interfering marks from injury or make-up, etc.), correlations of the measurements taken (in either 2D or, with improved outcome, in 3D) with those from a similar face are frequent [21]. In addition to running accidental errors, the use of facial recognition against deliberate fraudulent morphing presents a separate set of unique challenges. The extensive psychological literature on face processing suggests a number of vulnerabilities that could be exploited by people wishing to deceive ID checkers (whether human or machine). One such example has been highlighted by Scherhag et al. 2017 [20] and is presented in Fig. 10. A standard algorithm would “accept” any of the three morphed image versions as sufficiently similar to any of the two originals. This deception method could be easily exploited by identity fraudsters by misusing the morphed image as “bridge” between the real and false identity.

The effectiveness of the border control authentication processes and procedures thus rely on the robustness of the technological solution being capable of detecting counterfeits and identify perpetrators.

### Use of biometrics technology in the context of travel and modern society

In addition to challenges regarding functionality and cost efficiency, the overall acceptability of to-be-deployed biometrics system depends on whether public concerns have been adequately addressed. Causes for an individual’s or society’s apprehension may include perceived or real intrusion of privacy (in either physical or data terms), and other unwanted (temporary or lasting) consequences resulting from using the system. Regulatory compliance does not equal social acceptance even if these are interlinked. Parameters that can influence a person’s acceptance of a given technology comprise their physical health, their mental disposition, trust in the authority in charge, confidence in the technology, level of stress, social and educational and cultural background, reason for travelling, personality and personal preferences, the way the technology is presented, availability and type of information about it, among others, and these may differ in weight as well as be variable over time.

## The persona impact assessment method

The ‘architecture’ for impact assessment typically consists of two main elements that can be labelled ‘framework’ and ‘method’. These are supplemented by e.g., guidelines, templates or questionnaires. A *framework* constitutes an “essential supporting structure” or organisational arrangement for something which, in PERSONA’s context, concerns the policy for impact assessment, and defines and describes the structure, principles and rules thereof. In turn, a *method*, which is a “particular procedure for accomplishing or approaching something”, concerns the practice of impact assessment and defines the consecutive and/or iterative steps to be undertaken to perform such a process in accordance with the framework. A method can be generic, or it might be tailored down to a specific context [24, 25].

**Fig. 11:**
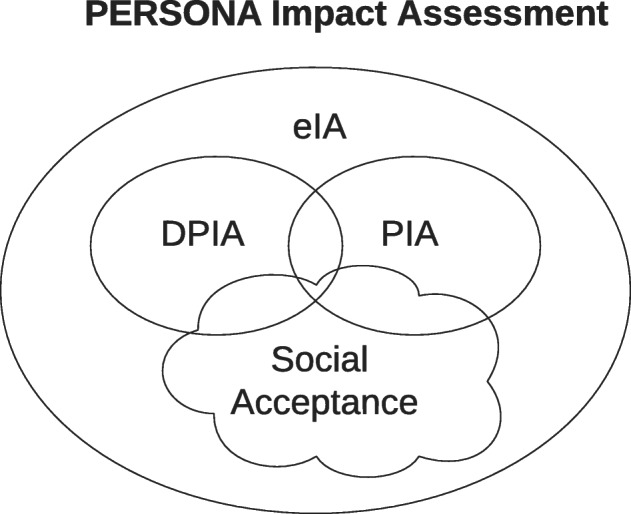
PERSONA integrated impact assessment method components [24]

While existing impact assessment methods enable evaluation of the performance and efficiency of a given biometrics technology, PERSONA project is novel in the way it seeks to address the public acceptance aspect. As visualised in Fig. 11, the resulting tailored and integrated assessment method will combine Ethical Impact Assessment (eIA) including social acceptance, Privacy Impact Assessment (PIA), and Data Protection Impact Assessment (DPIA). It will act as a tool which can inform decision-makers’ choices about related future technology deployments, and which can assist industry in designing appropriate product.

Funded under the European Union’s H2020 program, the multi-lateral PERSONA consortium comprises experts from the fields of engineering, social science, law, and end-users (law enforcement agencies and ministries) from nine different countries. Perhaps specific to the field of border security, acting authorities are by definition required to prioritising national interest over that of individuals or groups in society, and regulation should jump in where these don’t overlap. The General Data Protection Regulation (GDPR) constitutes an example for such regulation [1].

Through a catalogue of identified criteria, the PERSONA impact assessment method guides the assessor through a series of considerations which will help the respective authority to judge the impact of the border crossing solution they are wishing to deploy, in terms of its compliance with regulation, its risk under ethical aspects and its acceptance to society. PERSONA spans the much needed bridge between the interests of data controller and data subjects, where awareness especially of the latter forms an important component. It also sparks dialogue between designers, deployers and the wider public. In its collaboration with other relevant H2020 EU projects, PERSONA has started to test-run its assessment method on real travellers, targeting to-be-piloted next generation technology which has been created by these sister projects, a process likely to trigger a reframing of the presumed expectations for said technology.

A key aspect of PERSONA’s work was undertaken by The Brussels Laboratory for Data Protection & Privacy Impact Assessments (d.pia.lab at Vrije Universiteit Brussel) in its capacity as consortium and project coordinator. It relates to the study of EU regulatory framework that falls within the scope of national security. In addition to the general framework agreed by all member states, the data protection law related issues are regulated at national level as well. Therefore, it is important to note that the implementation of the no-gate crossing should consider the appointment of a data controller who is responsible for identifying the right legal framework applicable, which could be: GDPR, Law Enforcement Directive (LED) for which it will still be necessary to refer to national rules implementing the Directive, or EUDPR. This is elucidated further in the following extract from PERSONA deliverable D3.1 [24, 25]:

- “Under the GDPR and Art. 39 EUDPR, in order to determine whether a process of DPIA is required by law, the envisaged initiative has to be examined against the following six criteria:

i *Criterion 1 – high risk*: at a most general level, the GDPR requires a process of DPIA to be carried out (a) for processing operations likely to present a high risk to the rights and freedoms of data subjects, taking into account four qualitative criteria, namely the nature, scope, context and purpos of the processing of personal data; and (b) for data processing operations involving new technologies which constitute a particular trigger for the assessment process [Art. 35(1)]. These criteria, however, are not further defined, but could include e.g., the processing of special categories of personal data or data relating to criminal convictions and offences, data related to security measures or biometric data (i.e. the nature of processing operations), the amount of data processed, the geographical reach and the number of people affected (i.e. scope), in publicly accessible areas (i.e. context), data for profiling or automated decision-making (i.e. purpose) [cf. Recital 91]. It is for the data controller to determine whether a risk is “high”, for which determination the controller is held accountable. High risk is the first criterion also for Art. 39 EUDPR.
ii *Criterion 2 – enumeration*: the GDPR and Art. 39 EUDPR foresee three types of data processing operations for which a DPIA is required due to their likeliness to present high risk to the rights and freedoms of data subjects. These are:

- “systematic and extensive evaluation of personal aspects relating to natural persons which is based on automated processing, including profiling, and on which decisions are based that produce legal effects concerning the natural person or similarly significantly affect the natural person”;
- processing, on a large scale, of special categories of data or of personal data relating to criminal convictions and offences;
- “systematic monitoring of a publicly accessible area on a large scale” [Art. 35(3)].

iii *Criterion 3 – positive enumeration by data protection authorities*: a national or regional data protection authority (DPA) is entitled to determine, for its own jurisdiction, further types of data processing operations for which a process of DPIA is required [Article 35(4)]
  In case of EUDPR, the European Data Protection Supervisor shall establish and make public a list of the kind of processing operations which are subject to the requirement for a data protection impact assessment.
iv *Criterion 4 – negative enumeration by DPAs*: the same authority may determine, for its own jurisdiction, other types of data processing operations for which a process of DPIA is *not* required [Article 35(5)]. Both lists, if they involve cross-border processing operations, are to be communicated to the European Data Protection Board (EDPB) for an opinion, for which the consistency mechanism applies [Article 35(4)–(6)].
  In case of EUDPR, the European Data Protection Supervisor may also establish and make public a list of the kind of processing operations for which no data protection impact assessment is required.
v *Criterion 5 – selected previous assessment processes*: unless Member States decide otherwise, for personal data processed in order to comply with a legal obligation [Article 6(1)(c)] or processed in a public interest [Article 6(1)(e)], on the basis of EU law or Member States law, which have been already assessed within an assessment process in the context of the adoption of that legal basis, the process of DPIA is no longer required, provided this other assessment process essentially satisfied conditions laid down in the GDPR [Article 35(10)].
  In case of EUDPR, where processing pursuant to point (a) [processing is necessary for the performance of a task carried out in the public interest or in the exercise of official authority vested in the Union institution or body] or (b) [processing is necessary for compliance with a legal obligation to which the controller is subject] of Article 5(1) has a legal basis in a legal act adopted on the basis of the Treaties, which regulates the specific processing operation or set of operations in question, and where a data protection impact assessment has already been carried out as part of a general impact assessment preceding the adoption of that legal act, the process of DPIA shall not apply unless that legal act provides otherwise.
vi *Criterion 6 – exemptions for specific professions:* in case the processing operation concerns “personal data from patients or clients by an individual physician, other health care professional or lawyer”, these operations are not considered to be on a large scale and hence the process of DPIA is not required [Recital 91].

If any of the first three criteria is satisfied, a process of DPIA is mandatory. Conversely, if any of the three last criteria (criteria 4 and 5 in case of EUDPR) are satisfied, a data controller is exempted from carrying out the assessment process.”

## Ethics by design and social acceptance of technology

Where exactly technological innovation and ethics meet is difficult to define [7]. Each new technological development suggests new sets of behaviours, risks, and uses, hence ethics scholars and governing bodies must examine whether the application of such technologies has consequences that fall outside existing normative frameworks [6]. The first level of the dilemma refers to the struggle between existing laws and novel technologies: to what degree do existing legal frameworks provide enough protection against the possible problems, risks, and dangers that may arise in the slipstream of new technological developments?

The second level of the dilemma refers to the fact that laws are not the only regulatory tool in existence [8]. There is a wide range of means and tools that are used in regulating social behaviour. Lawrence Lessig, for example, in his well-known work *Code and Other Laws of Cyberspace* [14] distinguishes four modalities that regulate human behaviour: law, social norms, market, and architecture (code). Economics as well as political consensus, public communication, and education contribute to a constructive regulation of the societal profile beyond the rule of law [12]. According to some scholars, technology itself is a regulatory tool of human social and moral behaviours (techno-regulation) [9]. And not all the regulatory sources – regardless of whether they are legal or other in nature – are necessarily democratic forces that enhance the equal protection of freedoms and fundamental human rights, but may constitute forms of injustice [11].

Applying these premises to the case of biometrics technology, PERSONA tries to find ethics responses operating on a twofold analytical vision of “by design” and “social acceptance”. Ethics by design means that public and ethical principles (i.e. personal data protection) are built into products and services from the earliest stage of development [13]. In this context, social acceptance becomes an extremely relevant tool in detecting the leverage power of a technology for affecting perceptions and people’s trust, a capability so strong that Sherry Turkle discusses the potentially circular effect of silencing human conversations as a damaging consequence of always being in a “technologically connected” condition [23].

## Conclusion

By approaching the evaluation of technology from a value-laden perspective in which the abstraction of ethics principles embraces the contingency of social acceptance, the PERSONA impact assessment method serves as a roadmap to establishing potential legal and societal risks, to clarify public accountabilities, and outline social sustainable expectations. This approach leads PERSONA social researchers to argue that ethics can be incorporated in the design process of state-of-the-art border security technology itself operating on different levels of implications:

The first level deals with enhancing trustworthy and privacy protection in the design of technology itself, for example by innovative encryption methods. An example is MAGNETO [15], another H2020 project in which MMV (MultiMedia and Visions Lab) acts as partner. MAGNETO looks into technologies and solutions which permit Law Enforcement Agencies to consistently process massive heterogeneous data in a more efficient manner to transform it into solid and court-proof evidence. Even though this project is not directly related to border crossing technology, the approach towards the assessment method elaborated under PERSONA has informed work of MMV team members in MAGNETO, especially in view of the link between encryption and user privacy.

A second level has to do with the multidisciplinary enhancement of the public engagement of science and technology. Neither ethicists nor citizens might be aware of how a specific technology could be both used and abused, whereas its developers may not be as well-read as desired with respect to legal and societal requirements. Dialog between technology providers, decision makers, and citizens will ensure crucial knowledge transfer that can channel transparency and the creation of public awareness which, in turn, empowers citizens to make an informed choice.

A last level of implication relates to the increasing need for newly defined “ethics in engineering”. In fact, statistic data suggests that on the deployer side, an often encountered problem with biometric applications is the associated high risk for initially undetected or unreported bias and discrimination. As an example, many new-generation biometrics-based systems, including those realising behavioural biometry, “learn” in their training phase from millions of labelled examples. Their complex form (e.g. in case of deep neural networks) ultimately makes it impossible even for the developer to exactly understand and follow the thus acquired decision making process and resulting classification(s). Even if the recognition performance has been proven robust via comprehensive tests, the lacking traceability of the path between input data and the output recommendation raises additional ethical and legal questions.

Technology researchers are keen to highlight that the responsibility of how their results are used lies with the controller, not the creator, yet a protocol for the consequently necessary dialogue between developer and controller has not yet been fully established. PERSONA’s work constitutes a major contribution to bridging two information/communication gaps: the first between developer and deployer and the second between deployer and the wider public whereby the deployer (i.e. end user) acts as the executing party of the PERSONA impact assessment, in a push for increased transparency and accountability of both, developers and deployers, to society.
